# A Remarkable Organisation

**Published:** 1918-04-13

**Authors:** 


### A Remarkable Organisation.

The Quarterly Paper of the Liverpool Council of Voluntary Aid is always interesting, for it is a conspectus of the mass of committees and institutions engaged in public health and allied activities. With the caution that charity organisation must not be allowed to make a fetish of organisation for its own sake, the growth of these activities can be studied without confusion of mind. The success of the Liverpool Council can be judged from the fact that for the first time it has been entrusted with a definite sum for distribution. Its advice has often been sought, but a precedent has been created by a gift of £1,000 from Messrs. Pennefeather and Co. for distribution among Liverpool war charities. The Council is beginning to wield the power of the purse. Welfare workers will be interested to know that the Ministry of Munitions has obtained an important ruling from the Board of Inland Revenue, whereby contributions to schemes approved by the Welfare and Health Section of the Ministry "may be written off as a working expense." This ruling applies to all controlled factories. Hospital managers will be equally interested in the arrangement made by the Council, whereby the Liverpool Probate Registry notifies the Council of the contents of wills in which local charities are mentioned. Two such wills, benefiting sixteen charities, have been notified since the Council's last report. The Quarterly Paper's notes on new legislation and Government Reports make it valuable as a work for filing and reference.

